# Virus Infection Induces Immune Gene Activation with CTCF-anchored Enhancers and Chromatin Interactions in Pig Genome

**DOI:** 10.1093/gpbjnl/qzae062

**Published:** 2024-09-23

**Authors:** Jianhua Cao, Ruimin Ren, Xiaolong Li, Xiaoqian Zhang, Yan Sun, Xiaohuan Tian, Ru Liu, Xiangdong Liu, Yijun Ruan, Guoliang Li, Shuhong Zhao

**Affiliations:** Key Laboratory of Agricultural Animal Genetics, Breeding and Reproduction, Ministry of Education, College of Animal Science and Technology, Huazhong Agricultural University, Wuhan 430070, China; Key Laboratory of Swine Genetics and Breeding, Ministry of Agriculture and Rural Affairs, Huazhong Agricultural University, Wuhan 430070, China; Key Laboratory of Agricultural Animal Genetics, Breeding and Reproduction, Ministry of Education, College of Animal Science and Technology, Huazhong Agricultural University, Wuhan 430070, China; Key Laboratory of Agricultural Animal Genetics, Breeding and Reproduction, Ministry of Education, College of Animal Science and Technology, Huazhong Agricultural University, Wuhan 430070, China; Key Laboratory of Agricultural Animal Genetics, Breeding and Reproduction, Ministry of Education, College of Animal Science and Technology, Huazhong Agricultural University, Wuhan 430070, China; Key Laboratory of Agricultural Animal Genetics, Breeding and Reproduction, Ministry of Education, College of Animal Science and Technology, Huazhong Agricultural University, Wuhan 430070, China; Key Laboratory of Agricultural Animal Genetics, Breeding and Reproduction, Ministry of Education, College of Animal Science and Technology, Huazhong Agricultural University, Wuhan 430070, China; Key Laboratory of Agricultural Animal Genetics, Breeding and Reproduction, Ministry of Education, College of Animal Science and Technology, Huazhong Agricultural University, Wuhan 430070, China; Key Laboratory of Agricultural Animal Genetics, Breeding and Reproduction, Ministry of Education, College of Animal Science and Technology, Huazhong Agricultural University, Wuhan 430070, China; Key Laboratory of Swine Genetics and Breeding, Ministry of Agriculture and Rural Affairs, Huazhong Agricultural University, Wuhan 430070, China; Life Sciences Institute, Zhejiang University, Hangzhou 310058, China; Key Laboratory of Smart Farming for Agricultural Animals, College of Informatics, Huazhong Agricultural University, Wuhan 430070, China; Engineering Research Center of Intelligent Technology for Agriculture, Ministry of Education, Huazhong Agricultural University, Wuhan 430070, China; National Key Laboratory of Crop Genetic Improvement, Hubei Hongshan Laboratory, Huazhong Agricultural University, Wuhan 430070, China; Key Laboratory of Agricultural Animal Genetics, Breeding and Reproduction, Ministry of Education, College of Animal Science and Technology, Huazhong Agricultural University, Wuhan 430070, China; Key Laboratory of Swine Genetics and Breeding, Ministry of Agriculture and Rural Affairs, Huazhong Agricultural University, Wuhan 430070, China

**Keywords:** Pig, Chromatin, CTCF, ChIA-PET, Immunity

## Abstract

Chromatin organization is important for gene transcription in pig genome. However, its three-dimensional (3D) structure and dynamics are much less investigated than those in human. Here, we applied the long-read chromatin interaction analysis by paired-end tag sequencing (ChIA-PET) method to map the whole-genome chromatin interactions mediated by CCCTC-binding factor (CTCF) and RNA polymerase II (RNAPII) in porcine macrophage cells before and after polyinosinic-polycytidylic acid [Poly(I:C)] induction. Our results reveal that Poly(I:C) induction impacts the 3D genome organization in the 3D4/21 cells at the fine-scale chromatin loop level rather than at the large-scale domain level. Furthermore, our findings underscore the pivotal role of CTCF-anchored chromatin interactions in reshaping chromatin architecture during immune responses. Knockout of the CTCF-binding locus further confirms that the CTCF-anchored enhancers are associated with the activation of immune genes via long-range interactions. Notably, the ChIA-PET data also support the spatial relationship between single nucleotide polymorphisms (SNPs) and related gene transcription in 3D genome aspect. Our findings in this study provide new clues and potential targets to explore key elements related to diseases in pigs and are also likely to shed light on elucidating chromatin organization and dynamics underlying the process of mammalian infectious diseases.

## Introduction

The three-dimensional (3D) organization and dynamics of chromatin play important roles in eukaryotic gene transcription and regulation [1]. To comprehensively investigate chromatin interactions on a genome-wide scale, two methods have been developed, chromatin interaction analysis by paired-end tag sequencing (ChIA-PET) [2] and high-throughput chromosome conformation capture (Hi-C) [3]. ChIA-PET employs antibodies against specific protein factors, such as CCCTC-binding factor (CTCF) and RNA polymerase II (RNAPII), to pull down the chromatin associated with the target factor complex. This approach allows the identification of all loci bound by the factor, resolving factor-mediated chromatin interactions with base-pair resolution. Previous studies have shown that CTCF is the primary insulator-binding protein at the boundaries of higher-order genome architecture [4–7]. Furthermore, as a master weaver of the genome, CTCF interactions are involved in establishing chromatin architecture [8–10]. The boundaries of CTCF-defined topologically associated domains (TADs) participate in the connections of topological elements like enhancer-to-promoter [11–13]. RNAPII, as a core member of the transcription complex, is responsible for gene transcription with long-range chromatin interactions [14]. Therefore, a global high-resolution map of functional chromatin interactions can be generated based on interested factors, providing insights into the principal organization and dynamics underlying genome architecture.

The domestic pig (*Sus scrofa*) is an important livestock species for food protein supply, with its own importance for biological study. Due to its close similarity to human and its potential as a biomedical model, the domestic pig holds great promise as a donor for alleviating the shortage of organs for human xenotransplantation. A deeper understanding of porcine genomic organization will contribute to clinical applications involving porcine-to-human xenotransplantation. Recently, gene-editing modifications, such as clustered regulatory interspaced short palindromic repeats/CRISPR-associated protein 9 (CRISPR/Cas9), have been used to culture piglets with inactivated viral sequences in their genomes [15,16]. However, infectious diseases in pigs pose a severe impact on bio-safety, representing the top-priority consideration in porcine-to-human applications. In addition, infectious diseases cause severe economically loss in swine industry. For instance, porcine reproductive and respiratory syndrome (PRRS) annually costs losses more than 600 million dollars in North America [17], and the immune mechanism underlying viral infections remains poorly understood. Another highly contagious lethal viral disease, African swine fever (ASF), causes extremely high mortality in pigs and has serious economic or social consequences for swine industry [18–20]. Given that 3D genome structure represented with chromatin interactions between functional chromatin elements is related to gene transcription during viral infection, it is essential to study the 3D organization and dynamics of the pig genome in the process of virus infection.

To extensively characterize the chromatin interactions between functional elements and the higher-order organization in the pig genome, we stimulated porcine macrophage 3D4/21 cells with polyinosinic-polycytidylic acid [Poly(I:C)], a synthetic analogue of double-stranded RNA (dsRNA) and a molecular pattern associated with viral infection, as an infection model. Then we used ChIA-PET method to extract chromatin interactions targeting CTCF and RNAPII proteins, and applied CRISPR/Cas9 method to validate the functions of specific chromatin elements. The results demonstrate that chromatin interactions involving enhancers and/or promoters within their target loci are extensively involved in immune responses during viral infections. Because 3D4/21 cells are derived from porcine alveolar macrophages, our results are expected to contribute to elucidating the mechanisms of viral infectious diseases such as the PRRS in pigs. Our results also reveal that chromatin dynamics have close correlations with immune functional regulations via Poly(I:C) mimic infection in pigs.

## Results

### Typical immune genes are highly up-regulated after Poly(I:C) induction

**Figure 1:**
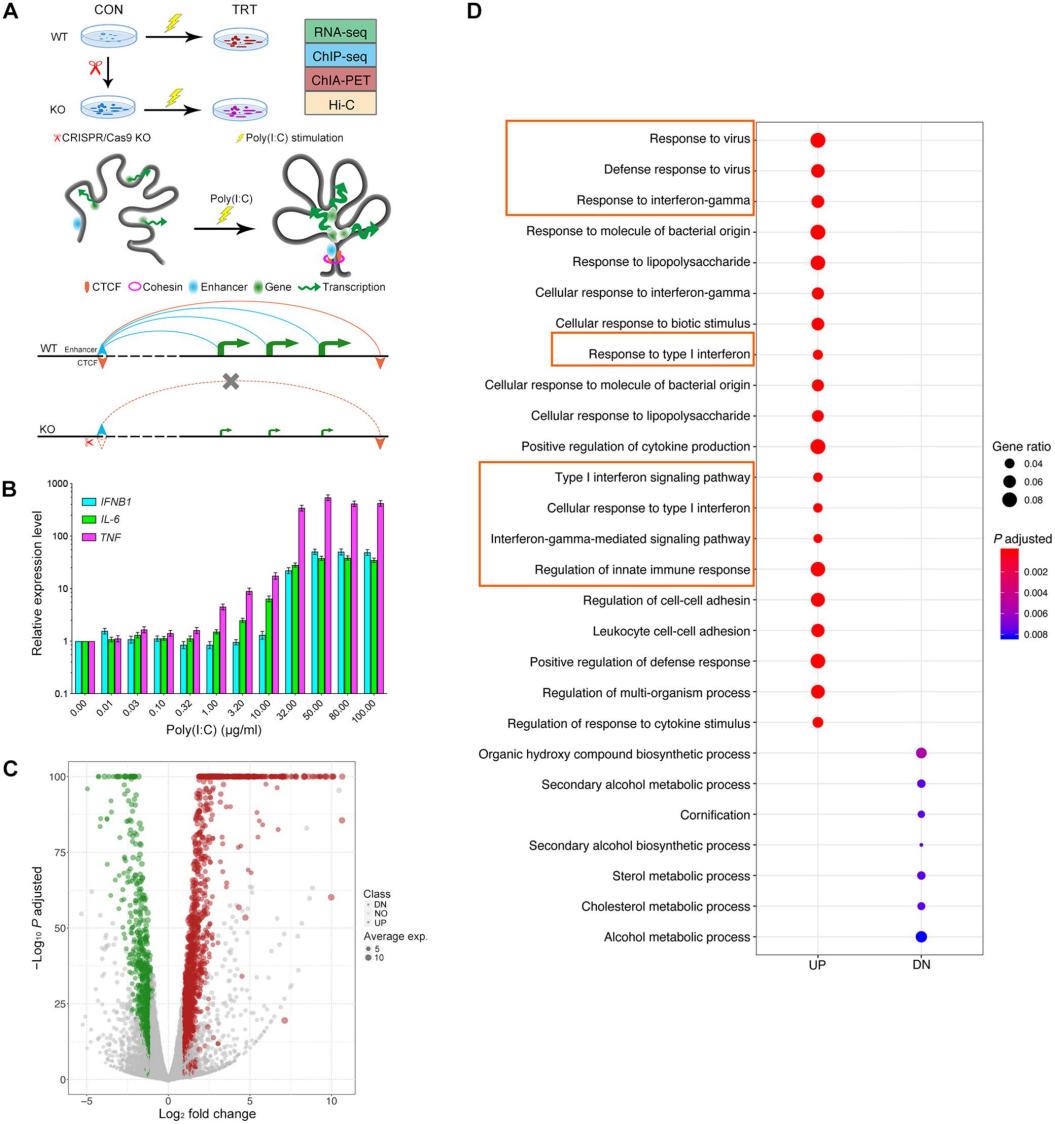
Characterization of gene transcription induced by Poly(I:C) in 3D4/21 cells **A**. Schematic diagram of this study. Top: cell processing experiment with scissors indicating CRISPR/Cas9 KO and lightning symbolizing Poly(I:C) induction. Middle: 3D chromatin conformation changes before and after Poly(I:C) induction. Bottom: cell transcription level changes after CRISPR/Cas9 KO. **B**. Dose-response analysis of Poly(I:C) induction in 3D4/21 cells. **C**. Volcano plot showing 1267 DEGs composed of 831 UP genes and 436 DN genes. **D**. GO enrichment analysis of DEGs. The immune-related terms that are effectively enriched in the UP genes (such as type I interferon, cytokine activity, and viral response) are marked by orange boxes. CON, control group; TRT, treated group; CRISPR, clustered regularly interspaced short palindromic repeats; Cas9, CRISPR-associated protein 9; KO, knockout; Poly(I:C), polyinosinic-polycytidylic acid; 3D, three-dimensional; UP, up-regulated; DN, down-regulated; NO, no significance; GO, Gene Ontology; DEG, differentially expressed gene; ChIA-PET, chromatin interaction analysis by paired-end tag sequencing; WT, wild-type; RNA-seq, RNA sequencing; ChIP-seq, chromatin immunoprecipitation followed by sequencing; Hi-C, high-throughput chromosome conformation capture; CTCF, CCCTC-binding factor; *IFNB1*, interferon beta 1; *IL-6*, interleukin 6; *TNF*, tumor necrosis factor.

Poly(I:C) is able to induce reactions in cells which can mimic immune responses to viral infection *in vitro*. In this study, we exposed porcine 3D4/21 cells to Poly(I:C) to simulate a viral infection *in vitro*. We used multi-omics techniques, including ChIA-PET and Hi-C, to investigate the regulatory elements of the functional genome during viral infection in pigs (**Figure 1**A). Our experiment showed that treatment with 50 μg/ml Poly(I:C) overnight was sufficient to significantly up-regulate the expression of the immune-related marker genes, such as *IFNB1* (Figure 1B). With RNA sequencing (RNA-seq), we detected 1267 differentially expressed genes (DEGs) (Table S1), including 831 up-regulated DEGs (UP) and 436 down-regulated DEGs (DN) (Figure 1C). Gene Ontology (GO) enrichment analysis showed that immune biological processes related to viral infection, such as type I interferon-related pathways and interferon-gamma-related pathways, were significantly enriched among the DEGs (Figure 1D).

### Epigenomic alterations mark gene activation after Poly(I:C) induction

**Figure 2:**
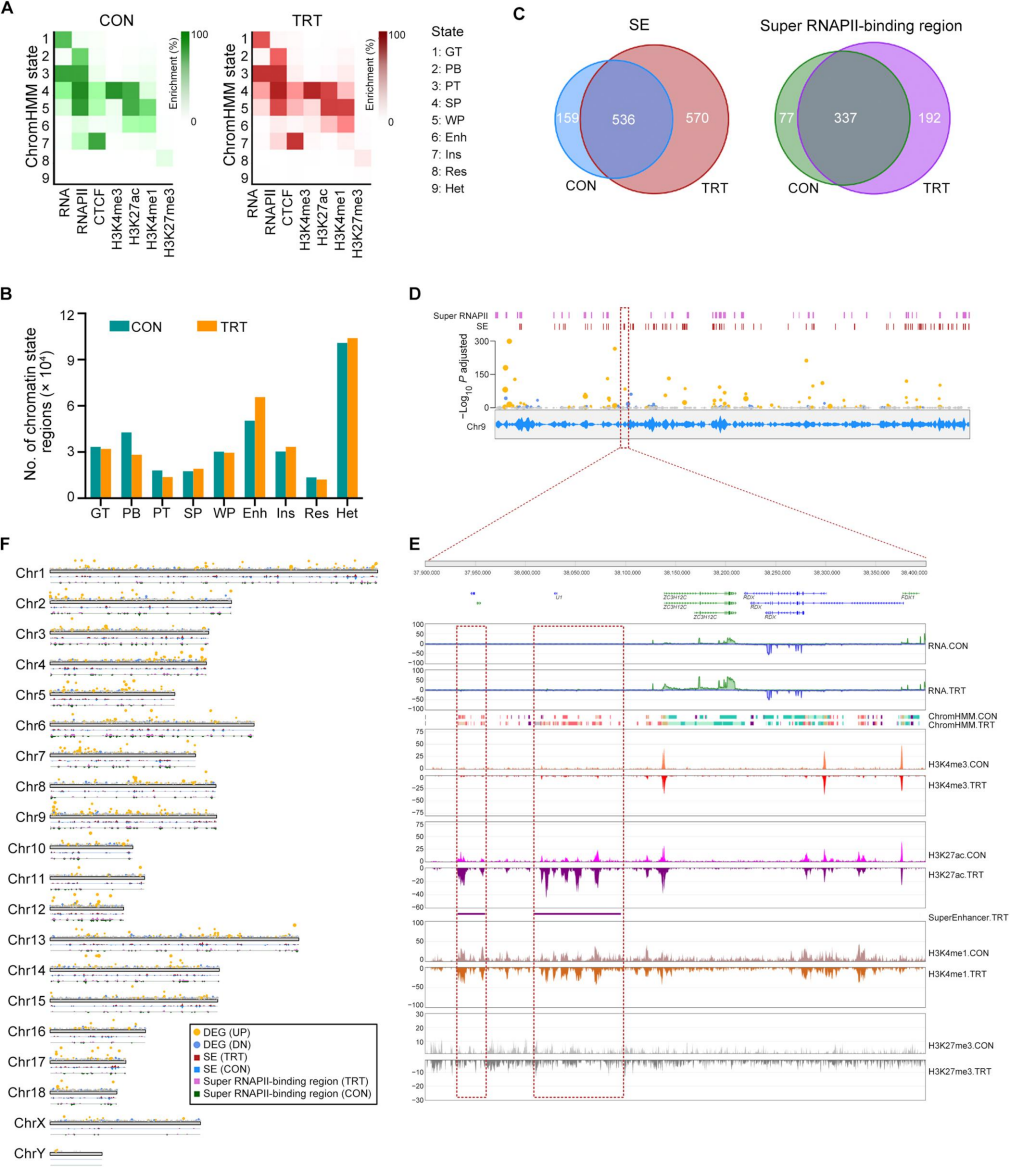
Epigenomic modification patterns before and after Poly(I:C) induction **A**. Heatmaps showing the distribution of nine ChromHMM chromatin states in CON (left) and TRT (right) cells separately assigned by four histone modifications (H3K27ac, H3K27me3, H3K4me3, and H3K4me1), CTCF-binding sites (CTCF), RNAPII-binding sites (RNAPII), and RNA transcription profiles (RNA). **B**. Distribution of chromatin state regions in CON and TRT cells. **C**. Venn diagrams showing the overlap of SEs (left) and super RNAPII-binding regions (right) between CON and TRT cells. **D**. UP genes correlated with SEs and super RNAPII-binding regions on Chr9. The DEGs are denoted as solid circles over the chromosomal ideogram (yellow for UP and blue for DN). The density curve of CECT-binding sites is shown inside the ideogram, and the peak locations of SEs and super RNAPII-binding regions were shown as bars above the ideogram. **E**. Example showing an inducible SE (located in Chr9:37,800,000–38,400,000) related with the activation of corresponding chromatin states and up-regulation of the neighbor gene (*ZC3H12C*). **F**. Co-localization of UP genes and super RNAPII-binding regions along chromosomes. RNAPII, RNA polymerase II; SE, super enhancer; GT, gene transcription; PB, RNAPII binding; PT, RNAPII transcription; SP, strong promoter; WP, weak promoter; Enh, enhancer; Ins, insulator; Res, repressor; Het, heterochromatin.

Epigenomic modifications play a crucial role in the regulation of gene transcription. By utilizing histone modifications (H3K27ac, H3K27me3, H3K4me3, and H3K4me1), CTCF-binding sites, RNAPII-binding sites, and RNA transcription profiles, we characterized the whole genome into 9 chromatin states: gene transcription (GT), RNAPII binding (PB), RNAPII transcription (PT), strong promoter (SP), weak promoter (WP), enhancer (Enh), insulator (Ins), repressor (Res), and heterochromatin (Het) (**Figure 2**A, Figure S1A and B). The total number of chromatin state regions increased from 296,821 to 329,801 after induction. The active histone modifications of those UP genes were significantly increased within the regions of gene body extended ± 3 kb, consistent with the increase in their transcription levels.

Notably, the number of typical enhancer states (characterized by enrichment of H3K27ac and H3K4me1) increased from 50,304 to 65,636 after induction (Figure 2B). Super enhancers (SEs) were identified based on the regions defined by enhancer chromatin states. We found 695 and 1106 SEs in over 20,000 typical enhancers in control (CON) and treated (TRT) cells, respectively (Figure S1C). A total of 536 SEs were shared between CON and TRT cells, while 570 SEs were specifically induced in TRT cells (Figure 2C; Table S2). Compared to the typical enhancers spanning 2 kb, the SEs exhibited a length exceeding 30 kb (Figure S1D; Table S2).

Similar to the SE definition, we also identified super RNAPII-binding regions by utilizing RNAPII-binding sites. These regions exhibited an expanded binding region exceeding 100 kb, contrasting to the narrow typical RNAPII-binding sites (∼ 0.7 kb) (Figure S1E and F). A total of 337 super RNAPII-binding regions were identified both in CON and TRT cells, while 192 super RNAPII-binding regions were specifically induced in TRT cells (Figure 2C; Table S3).

Interestingly, the UP genes were found to be clustered and highly colocalized with SEs and super RNAPII-binding regions, suggesting a close correlation between up-regulated transcription and SEs/super RNAPII-binding regions upon induction (Figure 2D). For example, the significant up-regulation of the UP gene *ZC3H12C* was associated with a nearby SE with an activated chromatin state (Figure 2E). The co-localization of UP genes and super RNAPII-binding regions suggests a potential correlation with enhanced transcription, possibly influenced by the coordinated activity of SEs in facilitating regulatory interactions between enhancers and promoters (Figure 2F).

### Transcriptional activation is associated with long-range enhancers

**Figure 3:**
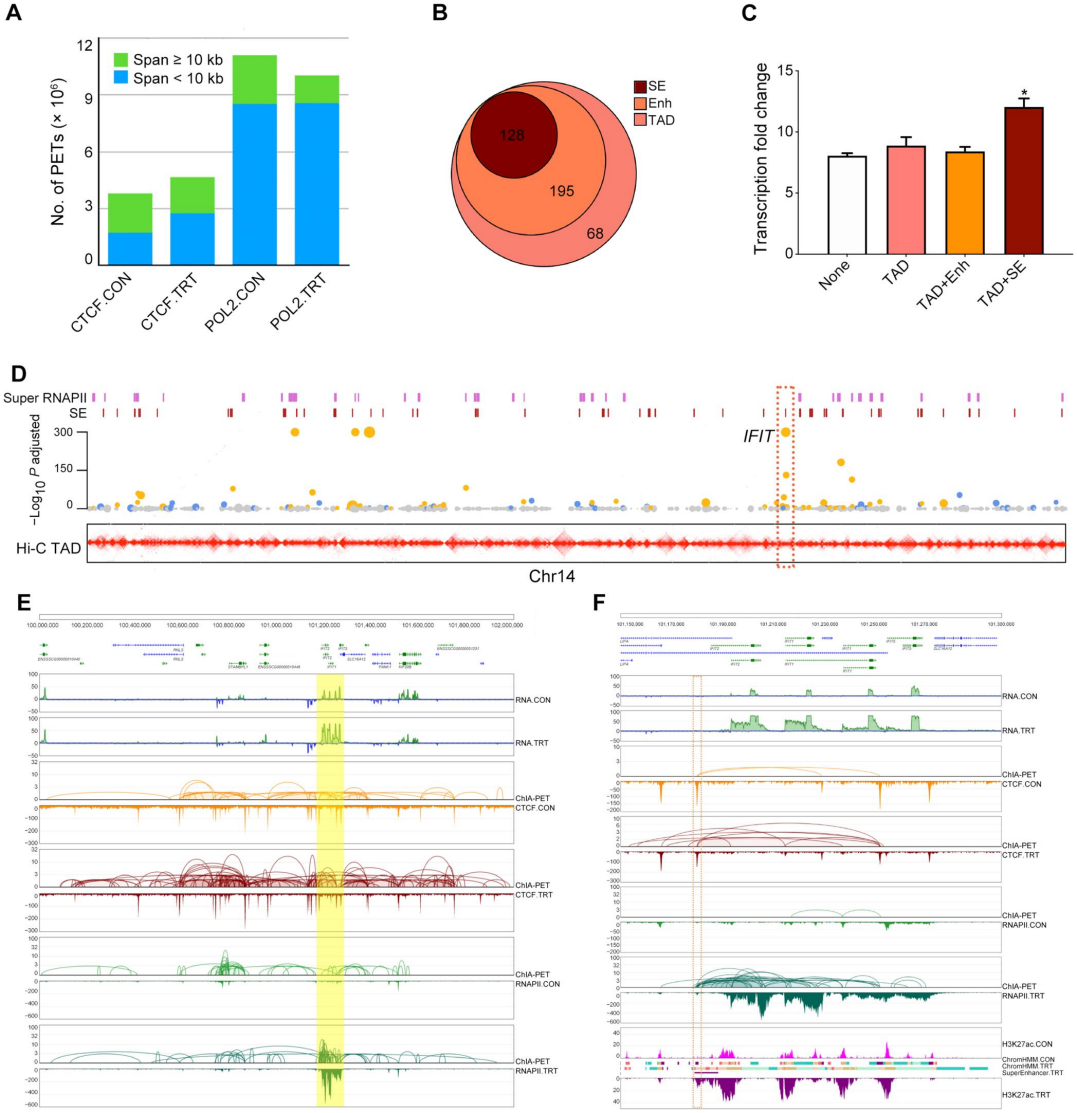
UP genes are associated with SEs via long-range interactions **A**. Number of CTCF- and RNAPII-mediated intra-chromosomal PETs in CON and TRT cells. **B**. Number of SE-possessing genes among the 391 UP genes located within TADs. **C**. The SE-possessing UP genes located within TADs had higher fold change of transcription levels. None represents UP genes not located within TADs; TAD represents UP genes located within TADs; TAD+Enh represents UP genes located within TADs that also contain enhancers; TAD+SE represents UP genes located within TADs that also contain SEs. **D**. The SEs and super RNAPII-binding regions located at TAD boundaries (bottom, contact map) were related with the UP genes on Chr14. The members of the IFIT gene family were indicated in the dashed box. **E**. The IFIT gene family (shadow box) was significantly activated by Poly(I:C) in TRT. **F**. The enhancer at Chr14:101,180,000 was located in a CTCF-mediated CCD (dashed box) and affected the transcription levels of *IFIT* genes. PET, paired-end tag; TAD, topologically associated domain; IFIT, interferon-induced protein with tetratricopeptide repeats; CCD, chromatin contact domain.

To gain a deeper understanding of the underlying mechanism of UP gene activation in immune response, we employed the ChIA-PET and Hi-C techniques to investigate chromatin interactions between enhancers and genes. ChIA-PET analysis effectively detected over 4 millions and 10 millions of CTCF- and RNAPII-mediated intra-chromosomal paired-end tags (PETs), respectively, in both CON and TRT cells (**Figure 3**A). Among them, about half of the CTCF-mediated intra-chromosomal PETs were in long-range interactions (55% for CON, 42% for TRT); however, only about one-fifth of the RNAPII-mediated intra-chromosomal PETs (23% for CON, 15% for TRT) were in the long-range interactions. These data demonstrate that the long-range chromatin interactions are significantly affected in both CTCF- and RNAPII-mediated chromatin organizations. According to the span distribution of PETs, we set a threshold of 10 kb to distinguish long-range interactions from self-ligations (Figure S2A–C).

To further dissect higher-order chromatin organization, we introduced the CTCF-mediated chromatin contact domains (CCDs) and RNAPII-associated interaction domains (RAIDs) to explain the relationship between DEGs and their regulations. The size and number of Hi-C TADs remained stable before and after Poly(I:C) induction (Figure S2D), implying that the CTCF-mediated CCDs within TADs had undergone substantial alterations. Although the sizes of CTCF-mediated CCDs and RAIDs exhibited minimal changes, the total number of CTCF-mediated CCDs increased (Figure S2E), indicating that the enhancers including SEs within CTCF-mediated CCDs could specifically up-regulate their target genes.

It was observed that among the 391 UP genes located within TADs, 128 UP genes processed SEs, and their average transcription level was significantly higher than that of other UP genes (Figure 3B and C). When integrating TADs from Hi-C data, the results further indicate that SEs and super RNAPII-binding regions are associated with UP genes, which are involved in long-range chromatin regulations through various regulatory elements, such as enhancers and transcription factors (TFs) (Figure 3D). For instance, the interferon-induced protein with tetratricopeptide repeats (IFIT) gene family was highly up-regulated, and the RAIDs identified from RNAPII ChIA-PET showed the chromatin interactions between the enhancer at Chr14:101,180,000 and all members of the IFIT gene family (Figure 3E and F). Additionally, TF-binding sites such as those around the *IRF1* and *STAT1* genes were enriched around the CTCF-binding site, likely involved in the establishment of the interactions between the enhancer and the IFIT gene family (Figure S2F).

### The enhancer at Chr14:101,180,000 activates *IFIT* genes via long-range chromatin interactions

**Figure 4:**
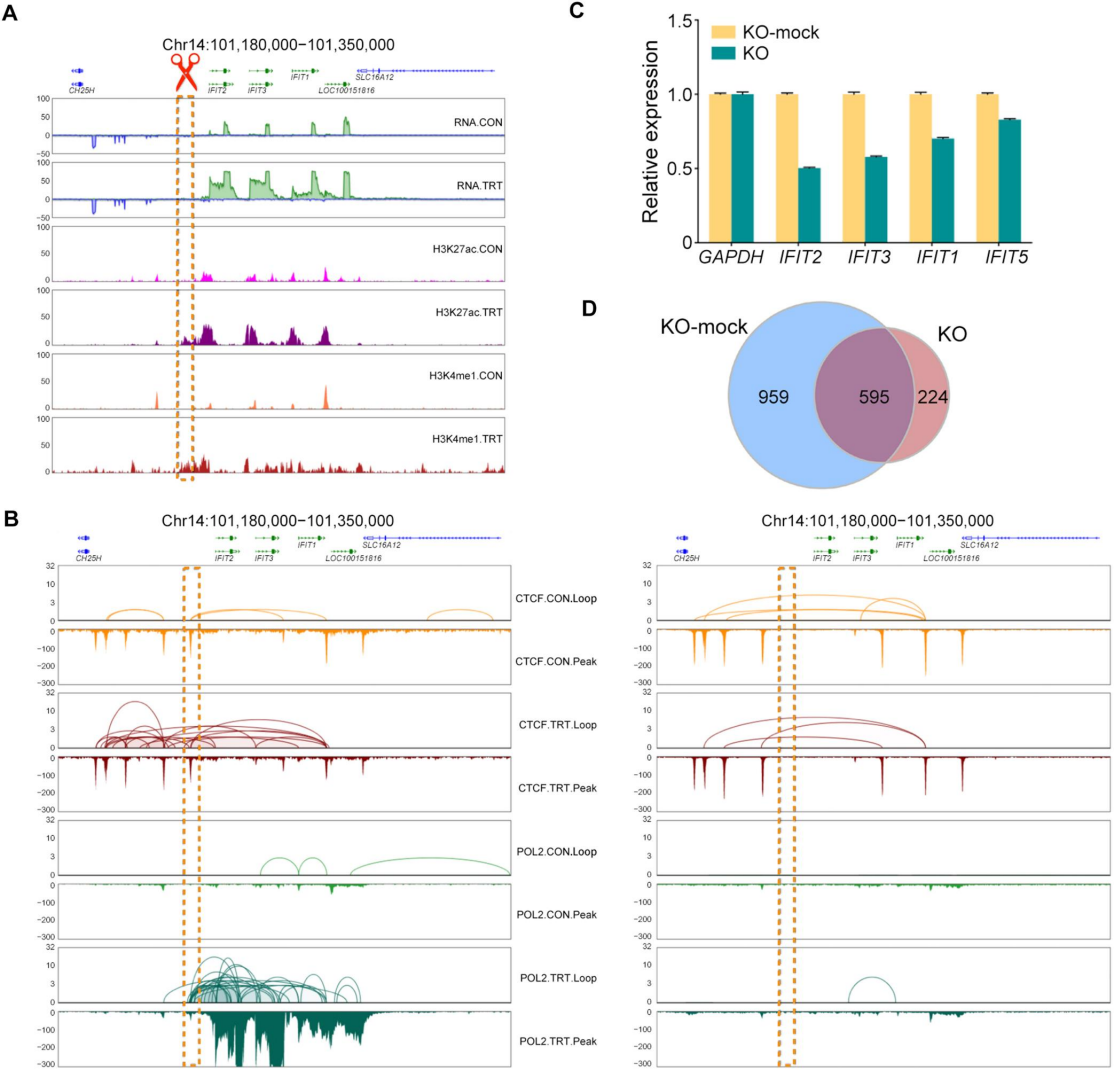
Enhancer interactions at *IFIT* locus validated by CRISPR/Cas9 KO **A**. The enhancer locus (dashed box) affecting the activation of *IFIT* genes located 13 kb upstream. **B**. In KO-mock cells, the enhancer locus (dashed box) was the boundary of CTCF-mediated CCD and RAID, and the CTCF loops anchored the enhancer and enhanced the transcription levels of remote *IFIT* genes (left panel). CRISPR/Cas9-mediated 167-bp deletion in the CTCF anchor within this enhancer locus depleted CTCF and RNAPII interactions (right panel). **C**. The transcription levels of *IFIT* genes were decreased in the homozygous KO cells (3D4/21^−/−^) compared with the KO-mock cells. The expression level of each gene in KO-mock cells was set to 1. *GAPDH* was used as a control. **D**. Venn diagram showing the overlap of DEGs [Poly(I:C) induction: before *vs*. after] between KO and KO-mock cells. RAID, RNAPII-associated interaction domain; *GAPDH*, glyceraldehyde-3-phosphate dehydrogenase.

The long-range enhancer at Chr14:101,180,000 was colocalized with a CTCF-binding site, which is thought to establish the CCD structure and dominate the activation of *IFIT* genes (Figure 4A). This site was selected for CRISPR knockout (KO) validation. Using the dual-single guide RNA (sgRNA)-mediated CRISPR/Cas9 KO system, the target sequences were deleted, and fluorescence-activated cell sorting (FACS) was applied to isolate positive cells. The homozygous KO cells were established by screening monoclonal cells (Figure S3A–C). Sequencing validation demonstrated the 167-bp deletion at this locus in the homozygous KO cells, which also led to the deletion of the CTCF-binding site there (Figure S3D).

To investigate transcription changes, the homozygous KO cells (3D4/21^−/−^) in both the CON and TRT groups were also subject to RNA-seq with three replicates (Figure S3E). As expected, the CTCF loops and CCDs via this locus were disrupted in the homozygous KO cells, leading to the disruption of the long-range RNAPII loops and RAIDs that originally enhanced the transcription of *IFIT* genes. This subsequently affected the transcription levels of the target genes (Figure 4B). The *IFIT* genes can still be activated by Poly(I:C) induction in the homozygous KO cells, but their relative expression levels were substantially reduced in the absence of the enhancer at this locus (Figure 4C, Figure S4).

When compared to the mock-treated cells, the KO cells lost 959 DEGs and gained 224 DEGs, excluding the 595 shared DEGs (Figure 4D). Additionally, another enhancer at Chr2:6,741,600 defined by CTCF on two well-known non-coding RNAs, MALAT1 and NEAT1, was also chosen for validation. A 75-bp fragment encompassing the CTCF-binding motif was deleted within this enhancer locus, leading to a notable decrease in the transcription levels of both the *MALAT1* gene and its partner *NEAT1* gene located 100 kb downstream (Figure S5).

### Single nucleotide polymorphisms reshape chromatin structure and affect the transcriptional differences of the GBP gene family

In recent years, the molecular mechanism underlying single nucleotide polymorphisms (SNPs) or quantitative trait loci (QTLs) in genome-wide association study (GWAS) have attracted much attention in swine [21]. The advent of 3D genome technology has provided a promising avenue to explore the regulation of target genes associated with SNPs identified in GWAS, particularly through long-range chromatin interactions. Previous studies have identified the *GBP* locus as a QTL for PRRS [22–24]. Notably, 36 SNPs distributed across 5 linkage disequilibrium (LD) blocks within this region were considered to be responsible for PRRS viral load and weight gain traits during infection (Figure S6A). However, the precise mechanism by which this region regulates the GBP gene family remains to be investigated.

**Figure 5:**
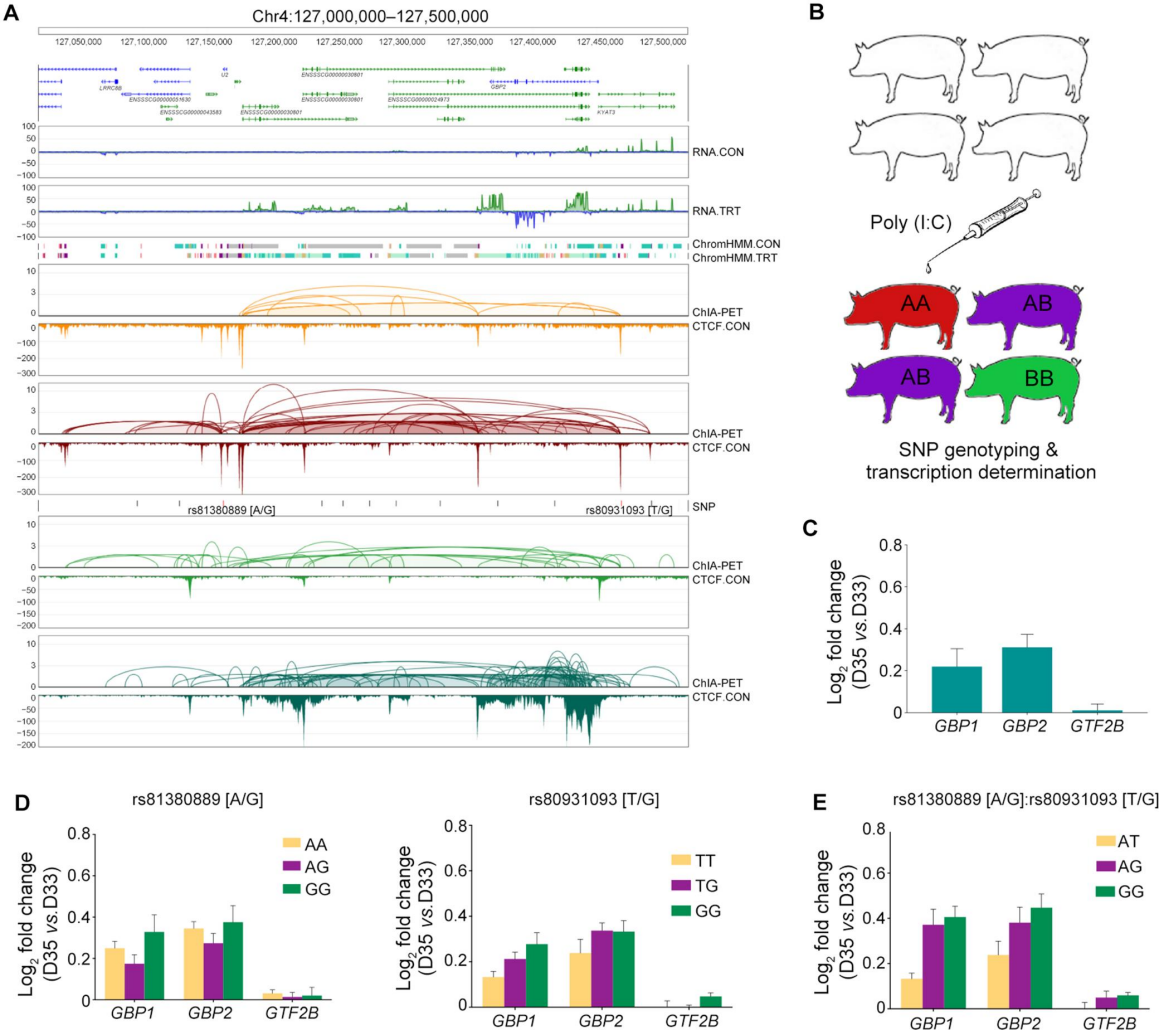
SNPs in ***GBP*** locus are associated with transcription alterations in pig population before and after Poly(I:C) induction **A**. The *GBP* genes located in the CTCF-mediated CCDs whose boundaries involved SNP variation of CTCF-binding motif (rs81380889). **B**. A flowchart for *in vivo* Ploy(I:C) induction. A total of 41 F_2_ piglets were injected with Poly(I:C) at 33 days old (D33), and then subjected to whole-genome SNP genotyping and transcription determination 2 days after injection (D35). **C**. Bar chart showing the increased transcription levels of *GBP1* and *GBP2* induced by Poly(I:C) injection in piglets at D35 *vs.* D33. *GTF2B* displayed no significant expression change before and after Poly(I:C) induction. **D**. Bar chart showing the varied transcription levels of *GBP1* and *GBP2* with different SNP variations (rs81380889 and rs80931093) at CTCF-mediated CCD boundaries. **E**. Bar chart showing that combination of haplotypes in two SNPs, rs81380889 and rs80931093, in the CTCF-mediated CCD can further decrease *GBP* transcription levels in Poly(I:C)-injected piglets. SNP, single nucleotide polymorphism.

In this study, the ChIA-PET and Hi-C data provided clear evidence that a TAD located in Chr4:126,600,000–127,600,000 plays a dominant role in determining the transcription patterns of the GBP gene family (Figure S6B). Upon Poly(I:C) induction, the transcription levels and histone modifications of all *GBP* genes were significantly up-regulated. Concomitant with the increase of CTCF loops, the RNAPII loops also increased dramatically upon induction, indicating that the rearrangements of CTCF-defined TADs in this region serve as a fundamental basis for the induced transcription of the GBP gene family (**Figure 5**A). Given that CTCF defines the boundaries of genomic architectures and provides the basis for the transcription complex of RNAPII focal bulbs, genes located around the boundaries, such as *GBP1* and *GBP2*, became more sensitive since they are closer to the variable genomic structures (Figure S6C). These findings further demonstrate that Poly(I:C) can effectively induce immune responses in pig individuals *in vivo*, similar to its effects observed in 3D4/21 cells *in vitro* (Figure 5B and C).

Interestingly, the transcription levels of genes within the sub-domain Chr4:127,150,000–127,538,000, specifically *GBP1* and *GBP2*, were found to be associated with the boundary genotypes and haplotypes (rs81380889 and rs80931093). In contrast, genes outside of the domain, such as *GTF2B*, exhibited independent transcription patterns regardless of the genotypes or haplotypes (Figure 5D and E). The rs81380889 SNP (G/A) is located within the CTCF-binding motif and plays a crucial role in maintaining the chromatin conformation that supports the transcription complex for the GBP gene family. The G-to-A mutation largely disrupted this transcription complex and decreased the sensitivity to induction, leading to the observed genotypic and haplotypic patterns of *GBP* gene expression.

## Discussion

In this study, we utilized porcine alveolar macrophages with Poly(I:C) induction as a model system to investigate the dynamics and molecular mechanism of distal regulation in the 3D genome of pigs. Our ChIA-PET data, obtained from this cellular model, reveal that CTCF-anchored enhancers reshape chromatin architecture in the pig genome, leading to the transcriptional activation of immune genes. To validate these findings, we conducted CRISPR/Cas9 KO experiments targeting a 167-bp CTCF-binding site at the *IFIT* enhancer locus, which significantly reduced the transcription levels of all *IFIT* genes. Furthermore, we found that a SNP (rs81380889) within the CTCF-binding site at the boundaries of TADs around the GBP gene family may play a crucial role in altering chromatin architecture and modulating immune responses triggered by PRRS virus in pig population.

As a fundamental component of chromatin infrastructure, CTCF plays a pivotal role in chromatin rearrangement [7], cell reprogramming [25], and differentiation [26] in the genomes of humans and mice. Our findings suggest that CTCF-anchored enhancers contribute to the transcriptional activation of immune-related genes and regulate chromatin quiescent states and dynamics in pig genome. Our data also extend the importance of RNAPII in chromatin structure maintenance and gene transcription through its role as part of the RAID and super RNAPII complexes to activate the transcription of immune-related genes, consistent with previous studies on transcription complexes [27–29]. We also observed that many SEs can be induced by Poly(I:C), indicating a close relationship between immune responses triggered by viral infection and SE establishment. Furthermore, we found that the majority of DEGs exhibited changes in KO cells compared to KO-mock cells both before and after Poly(I:C) induction. It may be due to the decreased expression levels of target genes in KO cells, which affects the expression of their associated genes and thus leads to changes in the entire gene regulatory network [30]. Finally, CTCF located at TAD boundaries has the ability to modulate chromatin architectures by the polymorphisms within its binding sites [31]. Allele-specific TAD structure alterations can influence imprinting genes like *H19* and *Dlk1* regions in mouse genome [32,33]. Our data provide similar evidence of haplotype alteration of TADs at the *GBP* locus in the F_2_ pig population.

The 3D genome architecture of pigs and its long-range regulation pattern explored in this study will facilitate future investigations in pigs. As a genetically modified model for viral infection, previous studies have well characterized the infection mechanisms of viral pathogens in pig diseases such as PRRS [34,35] and classical swine fever (CSF) [36,37]. Additionally, pigs may serve as medical models and xenotransplantation donors [15,16,38,39], making our data valuable for better understanding human health in terms of infectious viruses such as severe acute respiratory syndrome coronavirus 2 (SARS-CoV-2). Furthermore, we validated the functions of distal CTCF-anchored interactions between remote enhancers and their target genes. Lastly, we provided a new mechanism of PRRS-related regions in terms of chromatin structure alteration that could explain functional SNPs or causative mutations identified in GWAS of the human genome.

## Materials and methods

### Cell culture and *in vitro* Poly(I:C) induction

The 3D4/21 cell line from American Type Culture Collection (ATCC; accession No. CRL-2843), derived from porcine alveolar macrophages, was incubated in Roswell Park Memorial Institute (RPMI) 1640 medium (Catalog No. A10491-01, Thermo Fisher Scientific, Waltham, MA) supplemented with 10% fetal bovine serum (Catalog No. 10099-141, Thermo Fisher Scientific) and 1% non-essential amino acids (Catalog No. 11140-050, Thermo Fisher Scientific) in 5% CO_2_ at 37°C. To determine the optimal concentration of Poly(I:C) (Catalog No. tlrl-pic-5, InvivoGen, San Diego, CA) for induction, 3D4/21 cells grown at log phase (70% confluence) in 6-well plates were treated with Poly(I:C) at 12 concentration gradients (0, 0.01, 0.03, 0.10, 0.32, 1.00, 3.20, 10.00, 32.00, 50.00, 80.00, and 100.00 µg/ml) overnight. The cellular immune response calculated by the activation levels via three typical immune genes (*IFNB1*, *IL-6*, and *TNF*) were detected by quantitative polymerase chain reaction (qPCR) under the internal calibration of *GAPDH*. Finally, the Poly(I:C) concentration at 50 µg/ml overnight was selected to ensure sufficient activation for immune response induction in the cells.

### *In vivo* Poly(I:C) induction

The F_0_ founders were established by 8 sires (American pig breed named Duroc) and 18 dams (Chinese indigenous pig breed named Erhualian). The founders were crossed to produce 51 F_1_ individuals composed of 13 males and 38 females. Finally, F_1_ individuals were crossed and reproduced F_2_ population of 368 descendants which received immune challenge assay. All of the F_2_ piglets were injected with 0.5 mg/kg (body weight) Poly(I:C) at 33 days old (D33). At D35, the whole blood samples were collected to determine the hematologic parameters (lymphocyte count, hemoglobin, white blood cell count, *etc.*) by Celltac MEK-8222K Hematology Analyzer, and the effectors of immune responses and the antibody titers for interferon alpha (IFN-α), interferon gamma (IFN-γ), interleukin 8 (IL-8), and interleukin 10 (IL-10), were also measured by enzyme-linked immunosorbent assay (ELISA). Moreover, the genotypes and gene expression profiles for F_2_ piglets were determined by Illumina PorcineSNP60v2 Genotyping BeadChip and Affymetrix GeneChip Porcine Genome Array, respectively. Finally, the immune data of 41 F_2_ piglets that included SNPs, gene expression profiles, and the phylotypic traits of IFN-γ were incorporated with ChIA-PET interactions to investigate the roles of CTCF-mediated CCDs in regulating the expression of the GBP gene family.

### Long-read ChIA-PET library preparation

Long-read ChIA-PET libraries were prepared following the protocol as previously described [40]. Briefly, 100 million cells were firstly subjected to dual crosslink using 1.5 mM ethylene glycol bis (EGS; succinimidyl succinate) (Catalog No. 21565, Thermo Fisher Scientific) at room temperature for 45 min, followed by 1% formaldehyde (Catalog No. 344198, Merck, Darmstadt, Germany) for 20 min. The reaction was quenched with 0.25 M glycine (Catalog No. 50046, Sigma-Aldrich, Darmstadt, Germany). Harvested cells were sequentially lysed using 0.1% sodium dodecyl sulfate (SDS) lysis buffer for 15 min and 1% SDS lysis buffer for 5 min at 4°C. Nuclei were collected and resuspended using 0.1% SDS lysis buffer on ice. Each 1 ml nuclei suspension was sonicated by ultrasonic cell crusher (Catalog No. VCX130, Sonics, Newtown, CT) at 34% amplitude for 6 min. Chromatin was isolated by centrifugation at 13,000 r/min for 10 min and the supernatant was subjected to chromatin immunoprecipitation (ChIP) using 100 µg ChIP-grade CTCF primary antibody (Catalog No. ab70303, Abcam, Cambridge, UK) and 200 µg ChIP-grade RNAPII primary antibody 8WG16 (Catalog No. MMS-126R, Covance, Princeton, NJ), respectively. The on-beads ChIP DNA was ligated with the biotinylated bridge linker after end-blunting and A-tailing. Proximal ligation DNA was obtained by decrosslinking using proteinase K (Catalog No. AM2548, Invitrogen, Carlsbad, CA) digestion at 55°C overnight. The DNA was then randomly fragmented by Tn5 transposon and digested for 5 min at 55°C. Effective fragments were enriched by streptomycin-coated M280 beads. The on-beads (M280) DNA was amplified and concatenated with Illumina sequencing adapters by no more than 15 cycles of PCR. The length of fragments in libraries was selected from 300 to 500 bp using a fully automatic nucleic acid fragment recovery system (Blue Pippin, Sage Science, Bolton, MA). Each library was loaded to Illumina HiSeq 2500 platform using the 2 × 150 bp mode to generate paired-end reads. ChIA-PET data analysis is detailed in File S1.

### Hi-C library preparation

*In situ* Hi-C assays were performed as previously described [41]. Briefly, 20 million cells for each sample were fixed in 1% formaldehyde for 10 min, followed by 2.5 M glycine to quench the reaction. Fixed cells were lysed in the buffer (10 mM Tris-HCl pH8.0, 10 mM NaCl, 0.2% IGEPAL CA-630) with protease inhibitor cocktail tablets (Catalog No. P8340, Sigma-Aldrich). Five million pelleted nuclei were resuspended in 0.5% SDS and incubated at 62°C for 10 min, followed by addition of 10% Triton X-100 to stop SDS. Then, 400 U MboI (Catalog No. R0147, New England Biolabs, Ipswich, MA) and its buffer were added to digest chromatin overnight. Following the end repair by Klenow large fragment (Catalog No. M0210, New England Biolabs) and the A-tailing with biotin-14-dATP (Catalog No. 19524-016, Life Technologies, Frederick, MD), chromatin was subjected to proximity ligation with 50 U T4 DNA ligase (Catalog No. M0202, New England Biolabs) overnight at 16°C. The crosslinked DNA was reversed by proteinase K (Catalog No. AM2548, Invitrogen) digestion at 55°C for 30 min, followed by incubation overnight at 65°C. After phenol-chloroform extraction and purification, 130 µl biotinylated DNA was subjected to sonication shearing by Covaris S220 and was recycled back by adding AMPure XP beads (0.6× to 1.1×) to limit the length range of fragments (Catalog No. A63881, Beckman Coulter, Brea, CA). The biotin-labeled products were pulled down by streptavidin Dynabeads (Catalog No. 65001, Life Technologies). After end repair and A-tailing (Catalog No. E7546, New England Biolabs), Illumina-compatible adaptors were ligated to the end of DNA fragments (Catalog No. E7645, New England Biolabs). Bead-bound DNA was amplified for 6–10 cycles using Illumina TruSeq primers. The libraries were size-selected to 300–500 bp using a fully automatic nucleic acid fragment recovery system (Blue Pippin, Sage Science) and sequenced on an Illumina platform using the 2 × 50 bp mode. Two Hi-C libraries were prepared as replicates for both the control group and the treatment group. Hi-C data analysis is detailed in File S1.

### Sequencing data processing and genomic mapping

First, read quality was assessed by FastQC (http://www.bioinformatics.babraham.ac.uk/projects/fastqc/), and the possible adapters were removed from each end of raw reads using Cutadapt [42]. The clean read pairs were filtered to exclude any read shorter than 20 bp. The remaining pairs were then mapped to the Ensembl pig genome [43] (Sscrofa11.1) using Bowtie2 [44]. Under the paired-end mapping mode, only those pairs aligned concordantly exactly once, termed as unique mapping pairs (UMPs), were kept for further analysis. To address duplication introduced by PCR in library preparation, potential duplicated pairs identified by identical mapping coordinates in the Sequence Alignment/Map (SAM) file were removed to generate de-duplicated pairs (DDPs) for subsequent data processing. Binary Alignment/Map (BAM) files and Browser Extensible Data (BED) files were processed by SAMtools [45] and BEDTools [46], respectively. Density curves, boxplots, and scatterplots were drawn by the ggplot2 package [47], while alluvial plots were produced by the alluvial package. Gene schematic networks were constructed by NetworkAnalyst [48]. Peak data were loaded and visualized with the Integrative Genomics Viewer (IGV) [49].

### Site-specific CRISPR/Cas9 KO assay

Two sgRNAs, 5′-sgRNA and 3′-sgRNA, at a specific target site (dual-sgRNA), were firstly evaluated for off-target scores by the CRISPROR tool [50]. These sgRNAs were simultaneously cloned into the ends of a ∼ 500-bp insert containing a *U6* promoter derived from pUC57-sgRNA plasmid (Catalog No. 51132, Addgene, Watertown, MA). The dual-sgRNA PCR products were purified using DNA extraction kit (Catalog No. 9762, TaKaRa, Osaka, Japan) and subsequently cloned into pGL3-U6-sgRNA-PGK-puromycin plasmid (Catalog No. 51133, Addgene) at the BsaI site. The pEGFP-C1-Cas9 construct was used to separately express the Cas9 enzyme and green fluorescent protein (GFP). The 3D4/21 cells were cultured to ∼ 70% confluent in 6-well plates (Catalog No. 3516, Corning, Corning, NY). Co-transfection was performed using 1.5 µg/well pGL3 construct carrying the two sgRNAs and 1.5 µg/well pEGFP-C1-Cas9 encoding Cas9 and GFP, using Lipofectamine 2000 (Catalog No. 116680, Invitrogen) according to the manufacturer’s instructions. After 24 h, the transfected cells were collected and subjected to FACS cell sorting (FACSAria III, BD Biosciences, San Jose, CA). The GFP-positive cells were seeded in a 24-well plate (Catalog No. 3524, Corning) to isolate and culture monoclonal cells. These cells were validated by site-specific PCR followed by sequencing, and the homozygous KO cells were retained. The 3D4/21 cells transfected with non-specific sgRNAs were used as blank controls, termed KO-mock cells. The homozygous KO cells and KO-mock cells were then cultured under the same conditions for downstream analyses. In addition, the cutting activities of single sgRNA on pX330 plasmid (Catalog No. 42230, Addgene) were also evaluated in 3D4/21 cells by transfecting 2.5 µg/well of the construct using Lipofectamine 2000. The genomic DNA was purified 2 days after transfection, followed by site-specific PCR at target sites. The PCR products were subjected to T7 digestion (Catalog No. 0302L, New England Biolabs) following the manufacturer’s instructions. All sgRNA spacer sequences are as follows: *IFIT*-5′-sgRNA (5′-GACGTAGTCTCATTTATTTC-3′), *IFIT*-3′-sgRNA (5′-GAATCTGCCCCGCCCATGAA-3′), *MALAT1*-5′-sgRNA (5′-GATGGGCACAACGTTCAATG-3′), *MALAT1*-3′-sgRNA (5′-GGCTCTCGCCGCGGAGTTTT-3′), Mock-5′-sgRNA (5′-GTCAGAGTCCTAGTCAGCGA-3′), and Mock-3′-sgRNA (5′-ATCGGTACGTACCTGAAGCT-3′).

### GO enrichment analysis

The orthologous gene conversion from pig to human was firstly performed with Ensembl BioMart service. The identity of porcine genes to target human genes, 90%, was set as minimal lower limit to filter out ambiguous conversions. To match the optimal orthologous genes in human, one-to-one homology type, high confidence of orthologs, and no less than 50% identical to targets were also considered as priority items. The background gene list was used for all expressed genes in this study. The GO analysis and tendency plot of lexical terms were performed by GOplot R package [51]. The terms used in this study were customized from GO, InterPro, Kyoto Encyclopedia of Genes and Genomes (KEGG), Simple Modular Architecture Research Tool (SMART), and Database for Annotation, Visualization and Integrated Discovery (DAVID) public databases. The z-scores in bubble plots indicate the increasing (positive) or decreasing (negative) tendency of a certain biological process. The negative logarithm of adjusted *P* value was assigned to Y-axis in the bubble plots and adjusted *P* value < 0.05 was set as the threshold for significant terms. Tendency of lexical terms and circle chord graph were also plotted by GOplot.

## Supplementary Material

qzae062_Supplementary_Data

## Data Availability

The raw sequence data reported in this study have been deposited in the Genome Sequence Archive [52] at the National Genomics Data Center [53], Beijing Institute of Genomics, Chinese Academy of Sciences / China National Center for Bioinformation (GSA: CRA008270), and are publicly accessible at https://ngdc.cncb.ac.cn/gsa.

## CRediT author statement

**Jianhua Cao:** Methodology, Writing – original draft. **Ruimin Ren:** Validation, Data curation, Visualization. **Xiaolong Li:** Software, Writing – review & editing. **Xiaoqian Zhang:** Data curation, Writing – review & editing. **Yan Sun:** Validation, Software. **Xiaohuan Tian:** Methodology, Validation. **Ru Liu:** Methodology, Validation. **Xiangdong Liu:** Investigation, Data curation. **Yijun Ruan:** Supervision, Project administration. **Guoliang Li:** Supervision, Project administration, Writing – review & editing. **Shuhong Zhao:** Supervision, Funding acquisition, Project administration. All authors have read and approved the final manuscript.
