# Supplementary material for: Virus Infection Induces Immune Gene Activation with CTCF-anchored Enhancers and Chromatin Interactions in Pig Genome
Source: Genomics Proteomics Bioinformatics. 2024 Sep 23;22(5):qzae062. doi: 10.1093/gpbjnl/qzae062 (PMC11725346; doi:10.1093/gpbjnl/qzae062)
Supplement: qzae062_Supplementary_Data [file qzae062_supplementary_data.zip › Supplementary material captions.docx]

**Supplementary material**

**File S1 Supplementary materials and methods**

**Figure S1 Epigenetic alteration involved in gene transcription inducibility**

**A.** Except for CTCF, the RNAPⅡ binding levels and histone modifications of UP genes were higher in TRT than in CON which related with the higher transcription levels in TRT. **B.** The enrichments of 9 chromatin states assignments had similar patterns in both CON and TRT. **C.** The SEs were identified by using the enhancers of chromatin states which ranked 21,260 and 25,350 H3K27ac signals from CON (left) and TRT (right), respectively. **D.** The SEs were over 30 kb in length (right panels) compared with ~ 2 kb in length of typical enhancers (left panels). **E.** Total 7440 and 7860 RNAPⅡ signals were used to call super RNAPⅡ in CON and TRT. There were 414 and 529 super RNAPⅡs in CON (left) and TRT (right), respectively. **F.** The super RNAPⅡ was possessed with super long range (100–200 kb) compared with ~ 0.7 kb narrow typical RNAPⅡ binding sites. TES, transcription end site; TSS, transcription start site.

**Figure S2 Characteristics of ChIA-PET data**

**A.** Distribution of PET span has similar patterns both in CON and TRT. The long-range interactions, 10 kb (dashed line indicated) to 1 Mb, were used in this study and the self-ligation were excluded. The CTCF super-range interactions (span > 1 Mb) were also included in the loop cluster generation step. **B and C.** After excluding self-ligation PETs, the loop span distribution of CTCF (**B**) and RNAPⅡ (**C**) concentrated from 10 kb to 1 Mb along with the increasing of PETs per loop. **D.** The TAD size (kb) estimations from Hi-C (up panel), CTCF ChIA-PET (middle) and RNAPⅡ ChIA-PET (bottom) were calculated based on the loops with Pets≥5 in CON (cyan) and TRT (orange). The distribution of Hi-C TAD size had no significant change. **E.** Total 3,057 Hi-C TADs (top), 8,677 CCDs (middle) and 2,659 RAIDs (bottom) showed peak distribution at minimum Pets = 5. There were more CCDs in TRT than CON and the RAIDs were opposite. **F.** On the enhancer loci of *IFIT* genes, the transcription factors, IRF1 and STAT1, were identified based on JASPAR binding motif database.

**Figure S3 Construction of homozygotic KO cells via dual-sgRNA CRISPR/Cas9 system**

**A.** Two customized plasmid constructs used in this study. Dual-sgRNA construct carries two sgRNAs which were independently transcribed by U6 promoter. **B.** The 167 bp nucleotides deleted at Chr14:101,179,954–101,180,120 by targeting two guide RNAs (green and cyan), which included a CTCF binding motif (orange). **C.** For the KO clones at *IFIT* locus, 23 selected cell clones were extracted genomic DNA and T7E1 was conducted on the target PCR products. The #20 clone was finally chosen to establish cell line in 3 alternative homozygotes (#7, #8 and #20). **D.** The NGS sequencing result showed that the *IFIT* locus was successfully deleted on two homologous chromosomes in the KO cells. Compared with the WT cells, the KO cells had no reads covered at the locus. **E.** The PCA clustering of RNA-seq samples showed that the KO and WT samples were distinct in CON and TRT conditions. sgRNAs, single guide RNA; PCR, polymerase chain reaction; NGS, next-generation sequencing; T7E1, T7 endonuclease; PC, principal component; PCA, principal component analysis.

**Figure S4 Validation of the enhancer interaction at *IFIT* locus with KO experiment using new cell clone**

**A.** A T7 endonuclease digestion assay was used to identify the B8 clone (highlighted in the box) from 8 clones using target PCR product. **B.** The KO cells were conducted to FACS sorting, and positive cells (depicted in green) were conducted to screen monoclonal cells. **C.** The expression levels of related genes were lower in KO cells than in wild type cells upon stimulation with Poly(I:C), consistent with our previous results. FACS, fluorescence-activated cell sorting.

**Figure S5 Anchor deletion of CCD resulted in transcription decreasing of genes within the domain**

**A.** Based on the strong RNAPⅡ mediated interactions between *MALAT1* and *NEAT1*, the KO locus (red scissor) was one of CCD anchors which organized two non-coding RNA genes (~ 100 kb in distance) into one TAD. **B.** The target PCR products from 13 cell clones were tested by T7E1 enzyme digestion and the #8 clone was chosen for further study. **C.** The transcriptions of long-range target gene (*NEAT1*) were significantly decreased in the *MALAT1*^−/^^−^ mutant cells. The WT and KO cells were subjected to Poly(I:C) stimulation and the ratios of gene activation were determined by qPCR. The data were represented as mean +/− standard error of mean. **D.** The KO location at *MALAT1* locus (Chr.2:6,741,490–6,742,034) includes the CTCF binding motif (red) and two sgRNAs binding sites, upstream (green) and downstream (blue), bound at the end of 75 bp KO sequence. qPCR, quantitative polymerase chain reaction.

**Figure S6 Characterization of F_2_ pig population inducted by Poly(I:C)**

**A.** The GWAS LD block was overlapped with the CCDs in this region (bottom). The rs80931093 SNP (red bar in dashed box) on boundary of the CCD altered chromatin architectures to tune transcription alteration of *GBP* genes. **B.** The 800 kb TADs, Chr4:126,700,000–127,500,000, covered the *GBP* genes (top panel). After Poly(I:C) induction, only the RAIDs (bottom panel) were induced compared with unchanged CCDs (middle panel). **C.** The enhancer locus (arrow) on the CCD was responsible for activating the *GBP* genes. GWAS, genome-wide association study; LD, linkage disequilibrium.

**Table S1 The list of all DEGs in this study**

**Table S2 Super enhancers in 3D4/21 cells in this study**

**Table S3 Super** **RNAPⅡ in 3D4/21 cells in this study**
