# Supplementary material for: Virus Infection Induces Immune Gene Activation with CTCF-anchored Enhancers and Chromatin Interactions in Pig Genome
Source: Genomics Proteomics Bioinformatics. 2024 Sep 23;22(5):qzae062. doi: 10.1093/gpbjnl/qzae062 (PMC11725346; doi:10.1093/gpbjnl/qzae062)
Supplement: qzae062_Supplementary_Data [file qzae062_supplementary_data.zip › File S1.docx]

**File S1 Supplementary materials and methods**

**Stranded-specific RNA sequencing library preparation**

Cells were seeded in 6-well plates (3516, Corning, Corning, NY) to growth log phage. Total RNA was isolated using RNeasy Mini Kit (74104, QIAGEN, Hilden, Germany) treated with DNase Ⅰ (79254, QIAGEN, Hilden, Germany) according to manufacturer’s protocol. RNA purity (A260/A280 ratio) was measured on NanoDrop 2000 (ND-2000, Thermo Scientific, Waltham, MA) and its integrity was checked using Agilent 2100 Bioanalyzer. The RNA samples simultaneously satisfied A260/A280 ratio > 2 and RNA integrity number (RIN) > 8 were eligible for library preparation. One μg total RNA from each sample was prepared according to manufacturer’s instructions (RS-122-2201, Illumina, San Diego, CA). The ribosomal RNA was depleted from total RNA prior to constructing library. Six stranded-specific RNA sequencing (RNA-seq) libraries including 3 replicates each for control and treatment group, respectively, were equally multiplexed for Illumina NextSeq 500 platform using 2 × 75 bp mode to generate 60–80 million paired-end reads for each library.

**Chromatin immunoprecipitation (ChIP) and library preparation**

Chromatin immunoprecipitation (ChIP) grade primary antibodies for H3K4me1 (ab8895, Abcam, Cambridge, UK), H3K4me3 (ab8580, Abcam, Cambridge, UK), H3K27ac (ab4729, Abcam, Cambridge, UK), and H3K27me3 (07-449, Millipore, Billerica, MA) were, respectively, coated onto Dynabeads Protein G (10009D, Thermo Fisher Scientific, Waltham, MA) in Tube Revolver (88881002, Thermo Fisher Scientific, Waltham, MA) at 4°C overnight. Before lysing cell pellets, incubating cells were resuspended with 10 ml lysis buffer 1 (50 mM 4-(2-hydroxyerhyl)piperazine-1-erhanesulfonic acid (HEPES), pH 7.5, 150 mM NaCl, 1mM ethylenediaminetetraacetic acid (EDTA), 1% Triton X-100, 0.1% sodium deoxycholate, 0.1% SDS, and proteinase inhibitors), rotating for 30 min at 4°C, spinning down the cells at 2500 rpm for 5min at 4°C. Cells were again resuspended in 30 ml lysis buffer 2 (50 mM HEPES pH 7.5, 150 mM NaCl, 1 mM EDTA, 1% Triton X-100, 0.1% sodium deoxycholate, 1% SDS), rotating for 15 min at 37°C to obtain nuclei. Nuclei were suspended in lysis buffer 1 and subjected for sonication. The chromatin was fragmented with Ultrasonic Processor (VCX130, Sonics, Newtown, CT) by pulsing on 20s, off 30s, amplitude 34% for 6min. Chromatin was cleared by centrifuging at 13,000 rpm for 15 min at 4°C. Supernatant was collected and pre-cleared by incubating with 150 μl G/A Dynabeads for 2 h at 4°C with rotation. Then we got 50 μl pre-cleared supernatant out and saved at −20°C as input and incubated the rest and Dynabeads combined with specific antibodies for 12–16 h with rotation at 4°C. To reverse the crosslink, protein–DNA complexes were washed three times with lysis buffer 1, twice with lysis buffer 3 (50 mM HEPES pH 7.5, 350 mM NaCl, 1 mM EDTA, 1% Triton X-100, 0.1% sodium deoxycholate, 0.1% SDS), once with LiCl buffer (100 mM HEPES pH 7.5, 250 mM LiCl, 1 mM EDTA, 0.5% sodium deoxycholate, 0.5% Nonidet P40 Substitute), and twice with Tris-EDTA (TE) buffer (10 mM Tris-HCl pH 8.0, 1 mM EDTA). Then we added 200 μl Elution Buffer (1% SDS in TE 8.0) to protein–DNA complexes shocked in Digital Heating Shaking Drybath (88880028, Thermo Fisher Scientific, Waltham, MA) at 800 rpm for 30 min at 65°C and collected supernatant into new 1.5 ml tube. We then added 200 μl 10 mM Tris-HCl to clear Dynabeads and removed to 1.5 ml tube and repeated again. Proteins were digested with 10 μl protein K overnight at 55°C. ChIP–DNA was extracted using phenol in MaXtract High Density tubes (129056, QIAGEN, Hilden, Germany). The DNA concentration was measured by Qubit 3.0 (Q33216, Invitrogen, Carlsbad, CA) and the ChIP efficiency was calculated. Total 250 ng ChIP–DNA was sheared by High Performance Sample Preparation System (S220, Covaris, Westford, MA) to get 100–500 bp chromatin fragments. The library preparation protocol was based on the NEBNext Ultra Ⅱ DNA Library Prep Kit from Illumina, and these DNA products were then subjected to size selection and paired-end sequencing using Illumina HiSeq 3000 platform. Eight libraries for two conditions (control and treatment) and four histone markers were equally multiplexed subjecting to 1 × 75 bp single-end mode or 2 × 150 bp paired-end mode sequencing on Illumina NextSeq 500 or HiSeq 4000 platform. The average raw reads were 50 million for each library and the same size of input DNA library was sequenced as genomic calibrations as well.

**Reproducibility analysis of sequencing data**

The reproducibility of the sequencing data was evaluated referring to previous mentioned method [1]. The scatter plots and Pearson’s correlation coefficients were generated based on the number of reads in bins with size 10 kb. A higher correlation coefficient indicates better reproducibility. The high correlation coefficients from the biological replicate data either in RNA-seq or in chromatin interaction analysis by paired-end tag sequencing (ChIA-PET) demonstrated good reproducibility in this study.

**RNA-seq data analysis**

The unique mapped reads and de-duplicated reads were subjected to call raw read counts by using GenomicFeatures, GenomicAlignments, and Rsamtools R packages [2]. The gene annotation files were retrieved from Ensembl gene annotation for pig genome assembly (Sscrofa11.1). Because of stranded-specific library preparation, the parameter settings (stranded, paired-end, and union mode) were used for summarization of raw counts. The differentially expressed genes (DEG) were calculated by DESeq2 [3] R package using raw counts as inputs. Meanwhile, the size-factor normalized counts and the regularized log (rlog) transformation counts were also obtained based on raw counts. The other supporting data including outlier test (Cook’s distance), fitting accuracy (dispersion estimates), and principal component analysis (PCA) of samples were comprehensively approached by following the package instructions. The heatmaps of DEG rlog transformation counts were created using by pheatmap R package with the Minkowski algorithm setting for genes (row) and samples (column) clustering. The expression levels of DEGs in two groups, control and Poly(I:C) treatment, were relatively displayed from low to high, in which the immune responsive DEGs were zoomed in to demonstrate the effects of Poly(I:C) induction on the cells.

**ChIP-seq data analysis**

The raw reads were inspected for quality using FastQC and were trimmed adapters by Cutadapt. Clean reads were mapping on pig genome Sscrofa11.1 using Bowtie2 and the unique mapping reads were kept for removing duplicates by Picard MarkDuplicates. Peak calling was performed by model-based analysis of ChIP-seq 2 (MACS2) [4] and narrow peaks were called for CCCTC-binding factor (CTCF) and all histone markers except for H3K27me3 (broad peak calling). ChIP-seq heatmaps were drawn by ngs.plot [5] R package. The binding motifs of transcription factors were retrieved from JASPAR [6]. Binding motif plots based on sequence similarity inference were drawn by WebLogo [7]. The chromatin states were characterized by ChromHMM [8] software using: (1) 4 histone modification data from ChIP-seq; (2) CTCF and RNA polymerase Ⅱ (RNAPⅡ) binding peaks from ChIA-PET; (3) gene transcription information from RNA-seq. The ChromHMM was run with default settings and segmented pig genome into 9 distinct states: gene transcription (GT), RNAPⅡ binding (PB), RNAPⅡ transcription (PT), strong promoter (SP), weak promoter (WP), enhancer (Enh), CTCF insulator (Ins), repressor (Res), and heterochromatin (Het). These 9 chromatin states were represented as different color bars along with the browser tracks. The fold enrichments of chromatin states were also calculated at 8 genomic regions including gene body, gene exon, gene transcription start site (TSS), RNAPⅡ binding/docking site, genomic insulator, CpG island, and genomic intergenic region. The color depth for each chromatin state (row) indicated the portion of each item’s (column) contribution. More saturation color means more important contribution of the item to that of chromatin state. Based on typical enhancers defined by chromatin states, the super enhancers (SE) were identified by searching for clusters of binding sites for typical enhancers using Rank Ordering of Sequence Enhancement (ROSE) [9] within 12.5 kb of one another to be stitched together. The super RNAPⅡ were defined similar to SE except for using PB in chromatin states within 10 kb stitch.

**ChIA-PET data analysis**

Long-read ChIA-PET data processing pipelines were performed as previously described with minority modifications [10]. Because of using Illumina Nextera Tn5 to prepare ChIA-PET libraries, the potential Nextera adapters were firstly cut at either end of raw paired-end reads. The clean reads were filtered to retain the bridge linker included ones which will subsequently be split into possible 2–4 parts. The longer parts (minimal length > 20 bp) at both sides of bridge linker will be chosen as paired-end tags (PET) for further analysis. Two parts (or anchors) of PETs were independently mapped to pig genome assembly and the unique mapped reads were extracted by SAM flags. The polymerase chain reaction (PCR) duplicated reads were excluded by Picard MarkDuplicates to generate de-duplicated reads. Last, two anchors of a PET were matched based on reads IDs and the PETs with both anchors uniquely mapped will be considered as effective ones for further analysis. The PETs were divided into inter-chromosomal (two anchors located at two different chromosomes) and intra-chromosomal (two anchors located in one chromosome) based on their chromosomal coordination. The genomic span of intra-chromosomal PETs was investigated on its distribution, and the minimal threshold 10 kb was chosen to decimate self-ligation (< 10 kb, derived from single ChIP DNA fragments) and inter-ligation (> 10 kb, representing the long-range interactions between two different DNA fragments). To evaluate the intra-chromosomal interactions between two loci, the intra-chromosomal PET anchors extended 500 bp along the pig reference genome at both loci were overlapped and merged into a cluster which generated a loop linking two regions. The loops were defined by the number of PET counts (PC) connecting two loci and loops only having one PC were also called singletons. Because of the PC in a loop reflecting the frequency of interactions between two genomic loci, the bigger PCs in loops represent the stronger interaction between two loci. Using deep sequencing, over millions of PETs (~ 30 M for CTCF and ~ 10 M for RNAPⅡ) and loops were generated by ChIA-PET in this study. We therefore set the PC cutoff of loops as 5 throughout the interaction analysis. The noise control in ChIA-PET data processing was performed in two aspects. One is setting higher PC cutoff, say 3 or 5, in loop generation that ensured the singletons and low confidence loops excluded in interaction analysis. The other one is restricting the distances of loops in the range of 10 kb – 1 Mb interval which would largely eliminate the self and random ligations in proximity regions around binding sites. The super long-range (> 1 Mb) intra-chromosomal interaction loops were separately analyzed for higher-order topological proximity in chromatin organizations. Hence, in order to define high reliable intra-chromosomal interactions in this study, the loops with PC > 3 [false discovery rate (FDR) < 0.05] and 8 kb < interval/distance < 1 Mb were set as criteria to obtain proper intra-chromosomal interactions in CTCF and RNAPⅡ ChIA-PET data. On the premise of chromosomal territory theory, the intra-chromosomal PETs contributed to establish higher-order intra-chromosomal conformation like transcription factory/foci. The inter-chromosomal PETs, otherwise, were helpful to construct inter-chromosomal territories in the whole genome. To study the roles of RNAPⅡ mediated chromatin organizations, the intra-chromosomal interactions were primarily investigated in our study.

**Hi-C data analysis**

Raw data had been processed by quality check, adapter removal, and mapping onto pig genome as mentioned before. The contact maps were generated by HiTC [11] and HiC-Pro [12] and the coordinate duplicates were removed before calling Hi-C compartments. A total of > 1.4 billion reads for each control and treatment group were processed with Juicer tools [13] and > 800 million Hi-C contacts were subjected to construct topologically associated domains (TAD) matrix with 100-kb resolution. In order to reduce data size, the threshold of minimum interaction distance was set to 10 kb. Different Hi-C libraries were normalized using iterative correction and eigenvector decomposition [14] (ICE). Two replicates for each condition were combined using interaction data followed by removing coordinate duplicates. There were > 50 million interactions with > 10 kb distance in each condition. In this study, we focused on the Hi-C TADs composed by intra-chromosomal interactions. The contact map was presented using Juicebox [15].

**Quantitative real-time PCR**

Total RNA was isolated from 3D4/21 cells using RNeasy Mini Kit (74104, QIAGEN, Hilden, Germany) treated with DNase Ⅰ (79254, QIAGEN, Hilden, Germany) according to the manufacturer’s protocol. The concentration and quality were assessed by the spectrophotometer (NanoDrop 2000, Thermo Scientific, Waltham, MA). One μg RNA were reversely transcribed to complementary DNA using the PrimeScript RT Reagent Kit with Genomic DNA Eraser (RR047A, TaKaRa, Osaka, Japan). Standard quantitative real-time PCR was carried out in the CFX Opus 384 Real-Time PCR System (CFX Opus 384, Bio-Rad, Hercules, CA) to measure relative expression. Expression of target genes was normalized to the expression of 18s RNA. Data for each gene were shown as the fold change of the mean of results for wild type cells. The relative expression of the target gene in different samples was determined by quantitative real-time PCR with implementation of the 2^−∆∆Ct^ algorithm [16].

**Reference**

[1] Li G, Ruan X, Auerbach RK, Sandhu KS, Zheng M, Wang P, et al. Extensive promoter-centered chromatin interactions provide a topological basis for transcription regulation. Cell 2012;148:84–98.

[2] Lawrence M, Huber W, Pages H, Aboyoun P, Carlson M, Gentleman R, et al. Software for computing and annotating genomic ranges. PLoS Comput Biol 2013;9:e1003118.

[3] Love MI, Huber W, Anders S. Moderated estimation of fold change and dispersion for RNA-seq data with DESeq2. Genome Biol 2014;15:550.

[4] Zhang Y, Liu T, Meyer CA, Eeckhoute J, Johnson DS, Bernstein BE, et al. Model-based analysis of ChIP-seq (MACS). Genome Biol 2008;9:R137.

[5] Shen L, Shao N, Liu X, Nestler E. ngs.plot: quick mining and visualization of next-generation sequencing data by integrating genomic databases. BMC Genomics 2014;15:284.

[6] Khan A, Fornes O, Stigliani A, Gheorghe M, Castro-Mondragon JA, van der Lee R, et al. JASPAR 2018: update of the open-access database of transcription factor binding profiles and its web framework. Nucleic Acids Res 2018;46:D260–6.

[7] Crooks GE, Hon G, Chandonia JM, Brenner SE. WebLogo: a sequence logo generator. Genome Res 2004;14:1188–90.

[8] Ernst J, Kellis M. Chromatin-state discovery and genome annotation with ChromHMM. Nat Protoc 2017;12:2478–92.

[9] Whyte WA, Orlando DA, Hnisz D, Abraham BJ, Lin CY, Kagey MH, et al. Master transcription factors and mediator establish super-enhancers at key cell identity genes. Cell 2013;153:307–19.

[10] Tang Z, Luo OJ, Li X, Zheng M, Zhu JJ, Szalaj P, et al. CTCF-mediated human 3D genome architecture reveals chromatin topology for transcription. Cell 2015;163:1611–27.

[11] Servant N, Lajoie BR, Nora EP, Giorgetti L, Chen CJ, Heard E, et al. HiTC: exploration of high-throughput “C” experiments. Bioinformatics 2012;28:2843–4.

[12] Servant N, Varoquaux N, Lajoie BR, Viara E, Chen CJ, Vert JP, et al. HiC-Pro: an optimized and flexible pipeline for Hi-C data processing. Genome Biol 2015;16:259.

[13] Durand NC, Shamim MS, Machol I, Rao SS, Huntley MH, Lander ES, et al. Juicer provides a one-click system for analyzing loop-resolution Hi-C experiments. Cell Syst 2016;3:95–8.

[14] Imakaev M, Fudenberg G, McCord RP, Naumova N, Goloborodko A, Lajoie BR, et al. Iterative correction of Hi-C data reveals hallmarks of chromosome organization. Nat Methods 2012;9:999–1003.

[15] Durand NC, Robinson JT, Shamim MS, Machol I, Mesirov JP, Lander ES, et al. Juicebox provides a visualization system for Hi-C contact maps with unlimited zoom. Cell Syst 2016;3:99–101.

[16] Livak KJ, Schmittgen TD. Analysis of relative gene expression data using real-time quantitative PCR and the 2^−∆∆Ct^ method. Methods 2001;25:402–8.
