# Supplementary figures and images for: Virus Infection Induces Immune Gene Activation with CTCF-anchored Enhancers and Chromatin Interactions in Pig Genome
Source: Genomics Proteomics Bioinformatics. 2024 Sep 23;22(5):qzae062. doi: 10.1093/gpbjnl/qzae062 (PMC11725346; doi:10.1093/gpbjnl/qzae062)

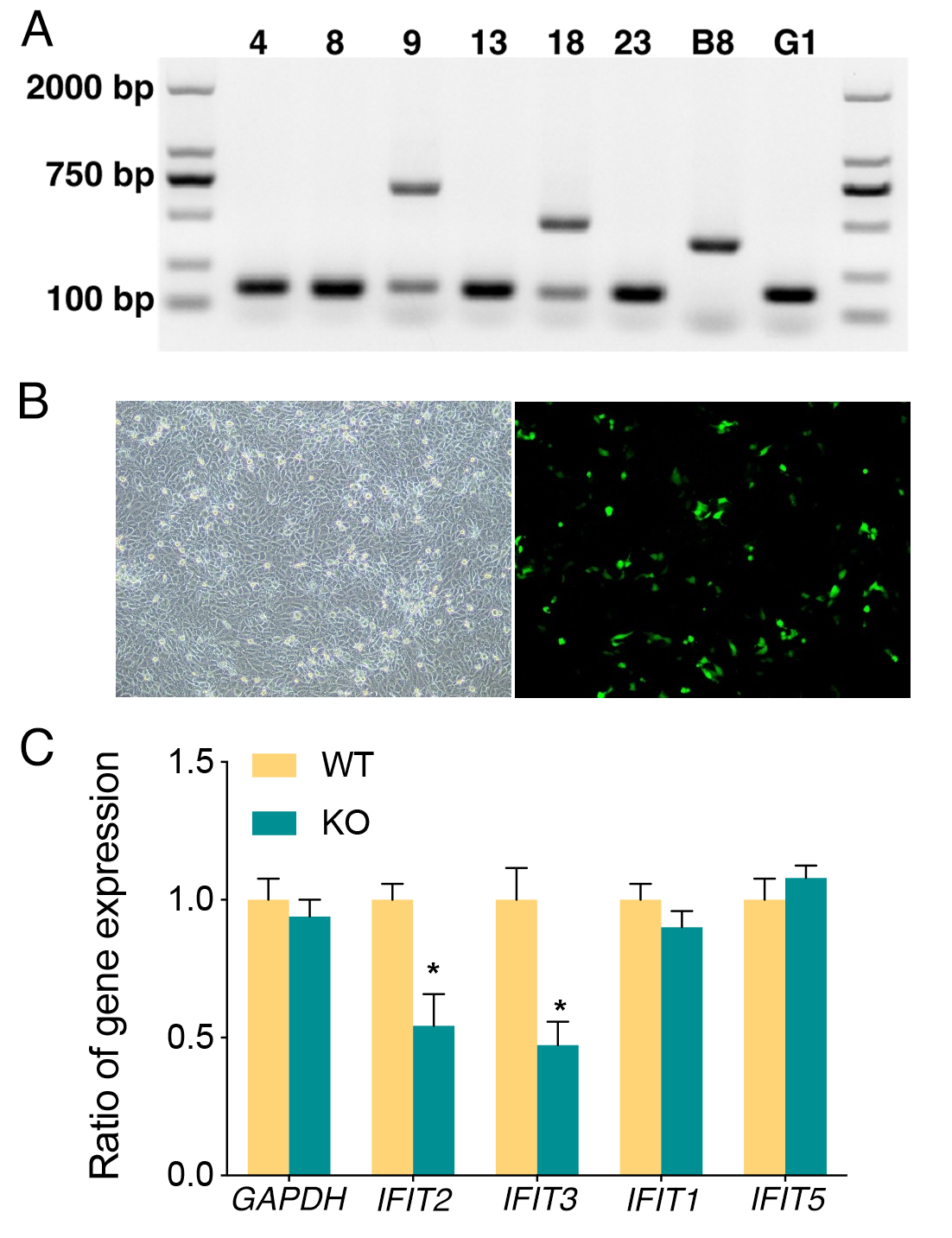

Supplement: qzae062_Supplementary_Data [file qzae062_supplementary_data.zip › FigS4.tif]

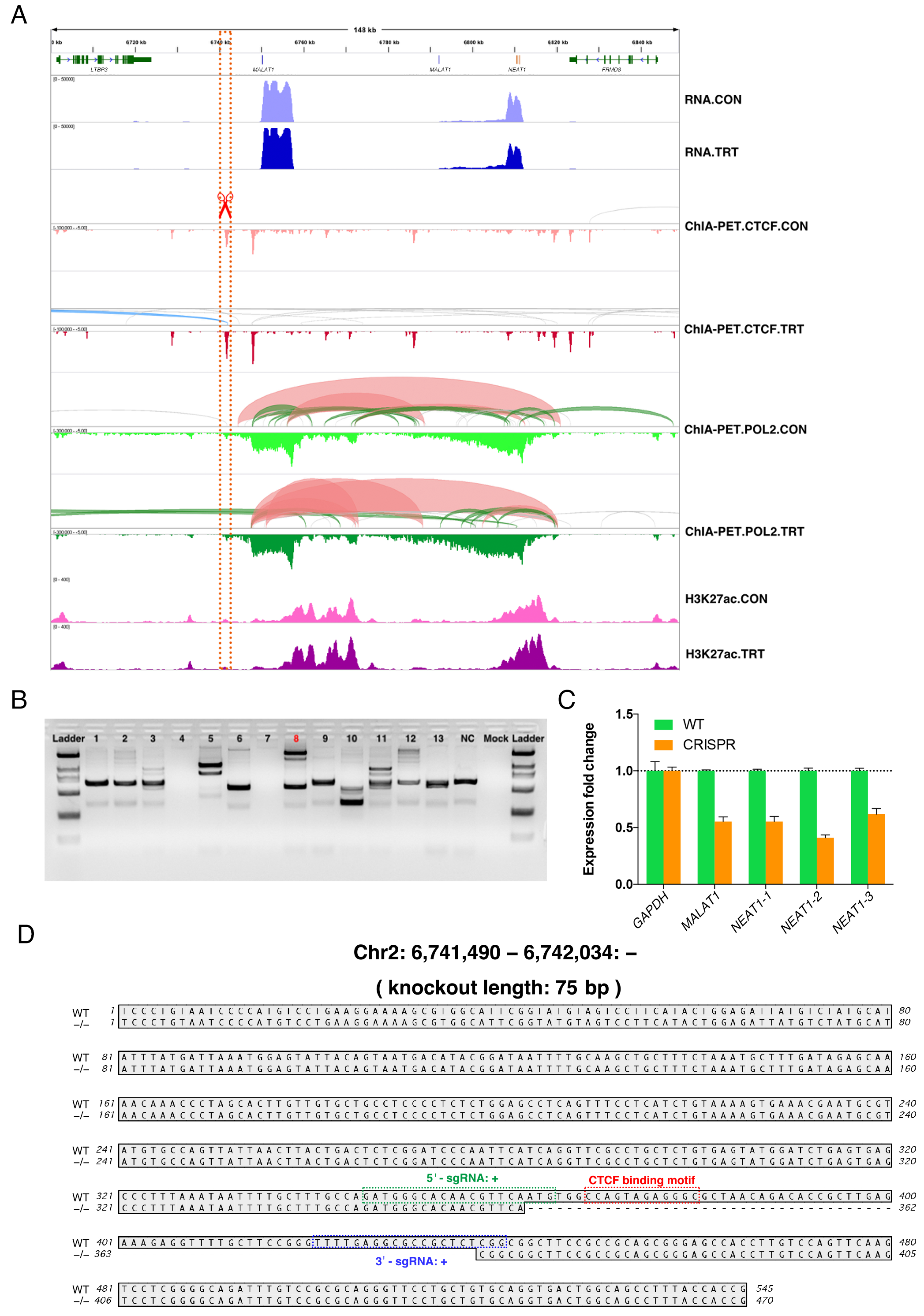

Supplement: qzae062_Supplementary_Data [file qzae062_supplementary_data.zip › FigS5.tif]

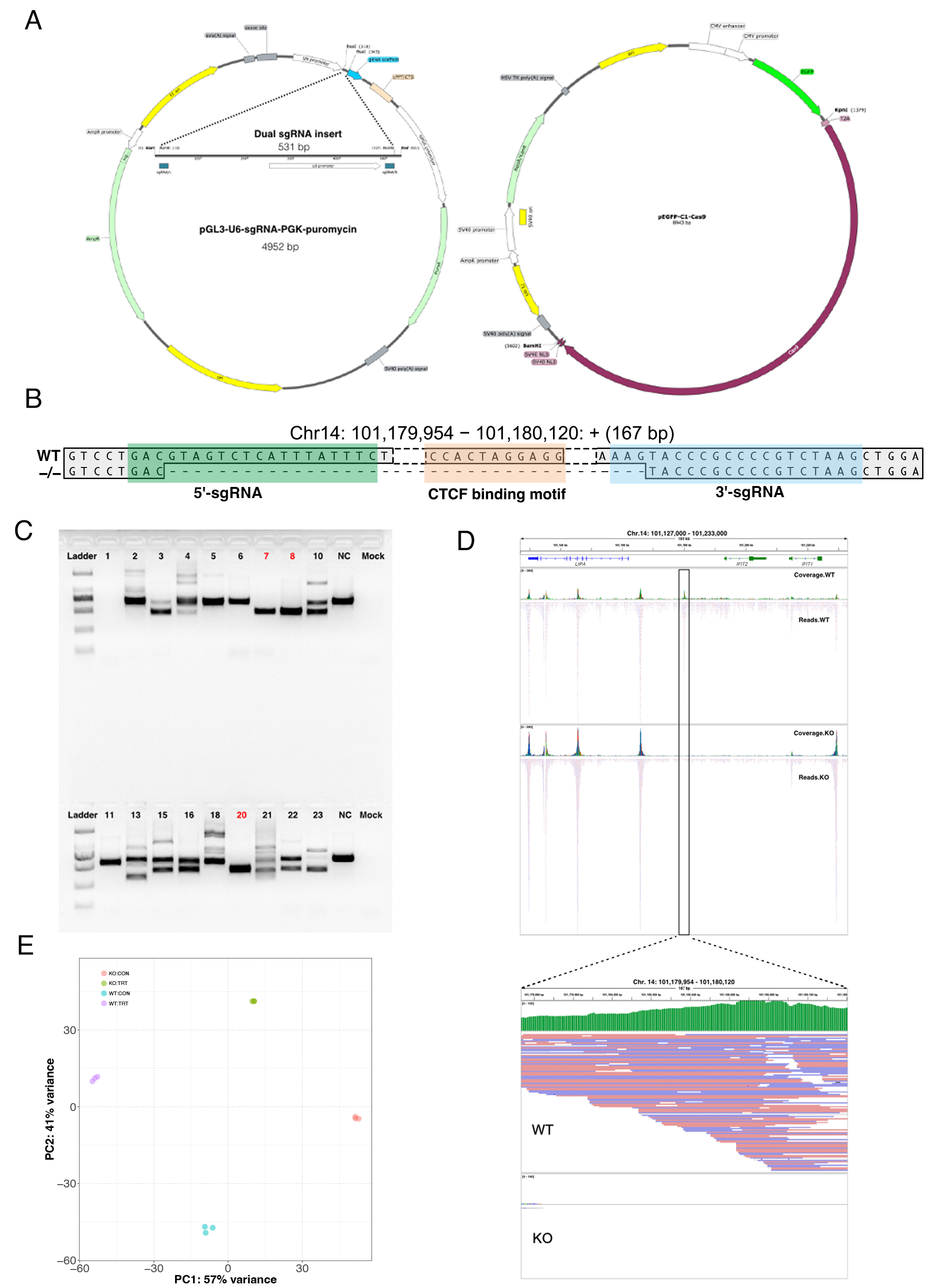

Supplement: qzae062_Supplementary_Data [file qzae062_supplementary_data.zip › FigS3.tif]

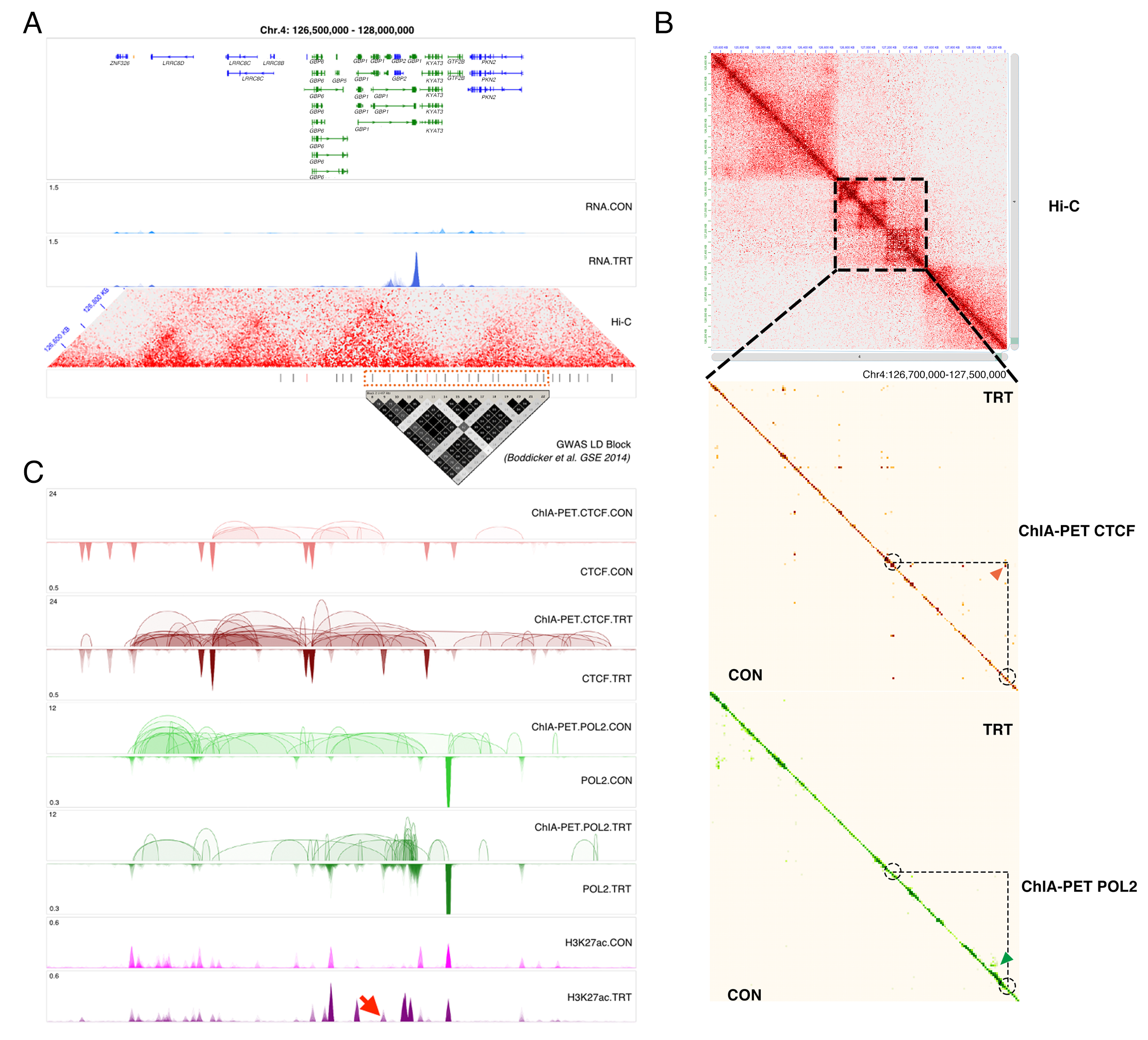

Supplement: qzae062_Supplementary_Data [file qzae062_supplementary_data.zip › FigS6.tif]

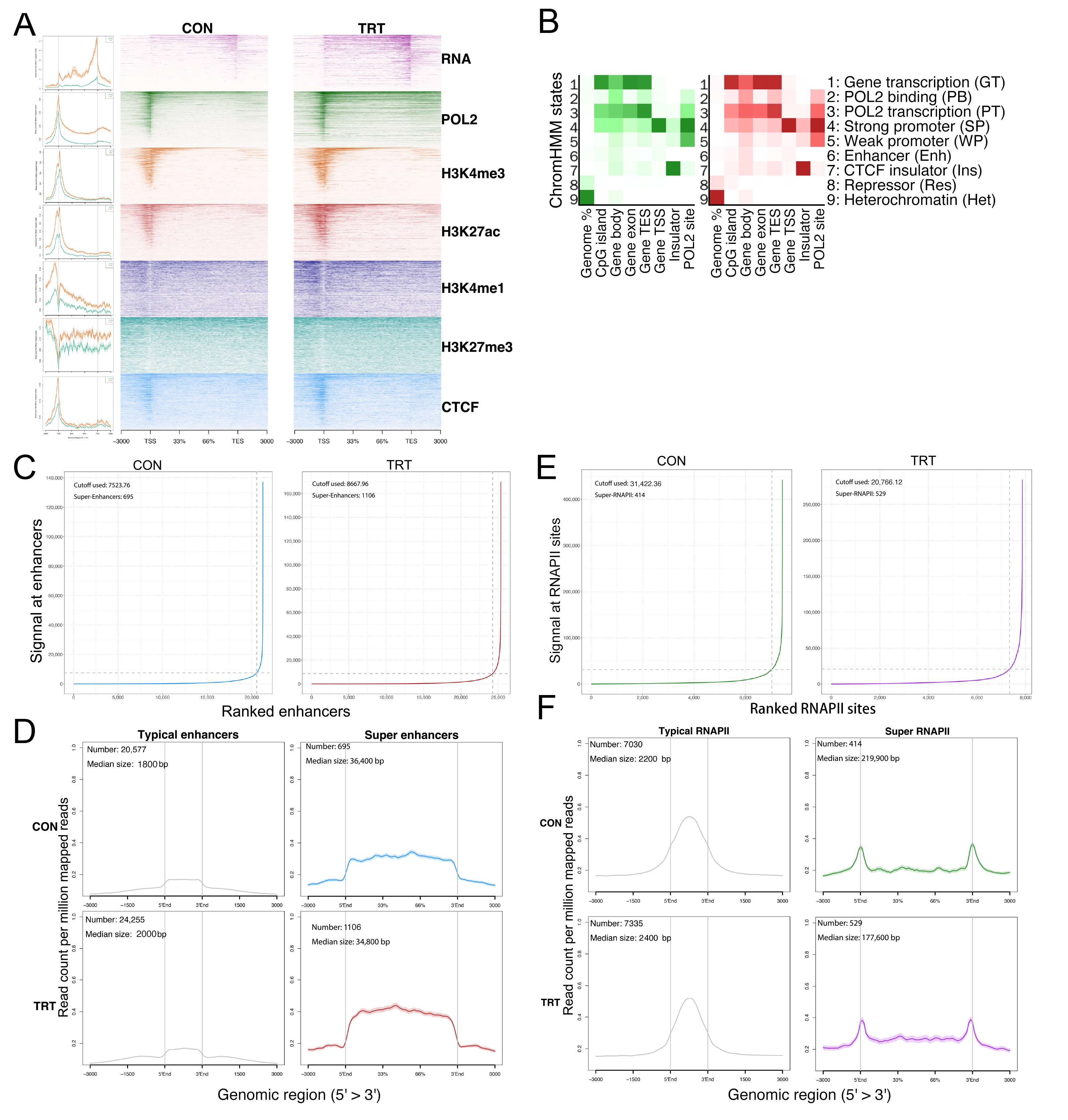

Supplement: qzae062_Supplementary_Data [file qzae062_supplementary_data.zip › FigS1.tif]

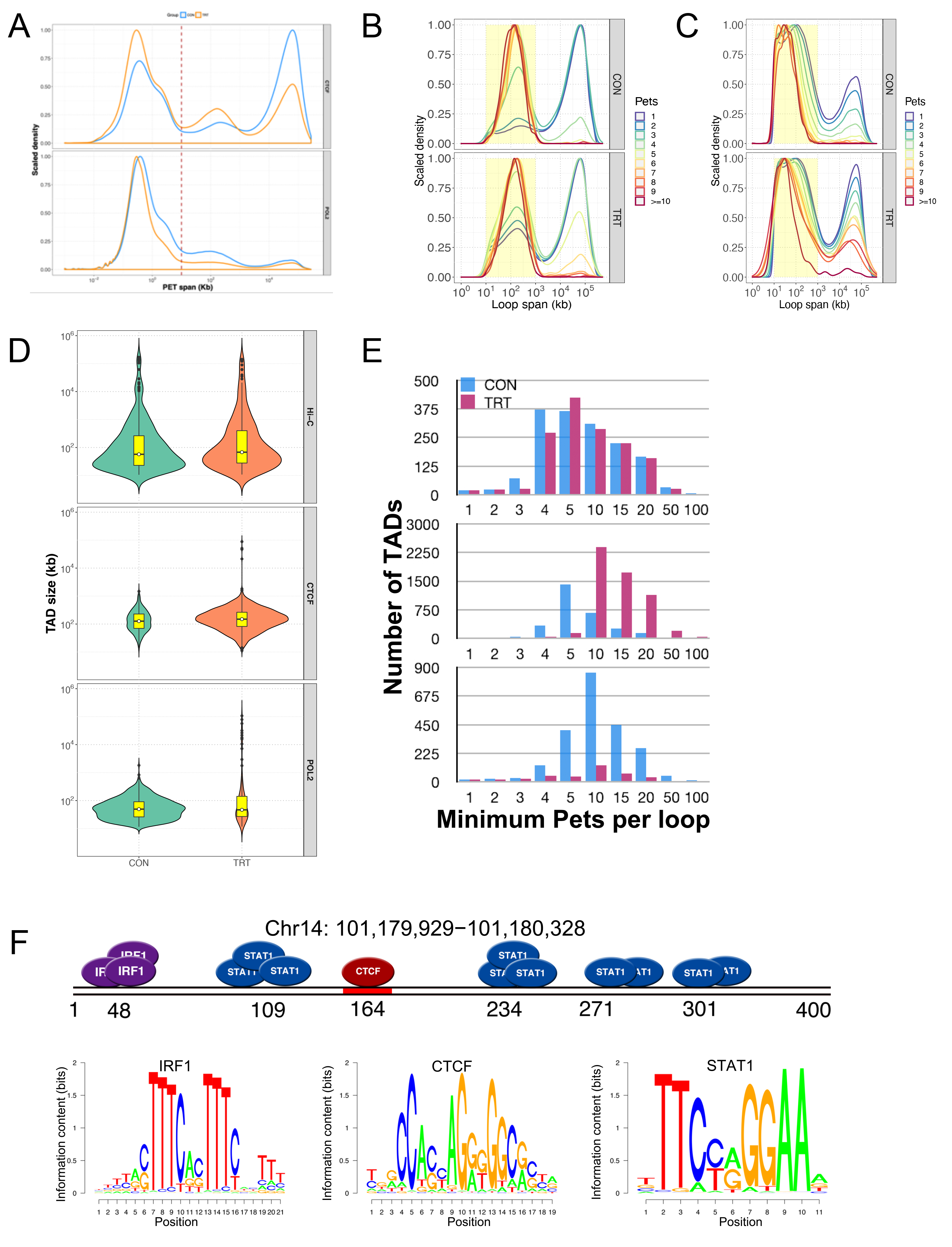

Supplement: qzae062_Supplementary_Data [file qzae062_supplementary_data.zip › FigS2.tif]
